# Water-Soluble Extract from *Actinidia arguta* (Siebold & Zucc.) Planch. ex Miq. and *Perilla frutescens* (L.) Britton, ACTPER, Ameliorates a Dry Skin-Induced Itch in a Mice Model and Promotes Filaggrin Expression by Activating the AhR Signaling in HaCaT Cells

**DOI:** 10.3390/nu11061366

**Published:** 2019-06-18

**Authors:** Wonwoo Lee, Yoonseon Jeong, Jong-Hyung Park, Chang Hyung Lee, Nayoung Yun, Doo Suk Lee, In-Jeong Nam, Jung-Dong Kim, Kee Dong Yoon, Miwon Son, Sunyoung Kim

**Affiliations:** 1 R&D Center for Innovative Medicines, Helixmith Co., Ltd., Seoul 08826, Korea; wwlee@helixmith.com (W.L.); ysjeong@helixmith.com (Y.J.); jhpark@helixmith.com (J.-H.P.); chlee@helixmith.com (C.H.L.); nyyun@helixmith.com (N.Y.); dslee@helixmith.com (D.S.L.); ijnam@helixmith.com (I.-J.N.); jdkim@helixmith.com (J.-D.K.); mwson@helixmith.com (M.S.); 2 College of Pharmacy, The Catholic University of Korea, Bucheon 14662, Korea; kdyoon@catholic.ac.kr

**Keywords:** ACTPER, itch, dry-skin, filaggrin, AhR

## Abstract

With a complex etiology involving multiple factors, the condition known as itch is a primary symptom of many skin diseases. Current treatment methods are ineffective for addressing itches caused by dry skin, for example. We developed a botanical extract, ACTPER, made from a mixture of *Actinidia arguta* and *Perilla frutescens*, which have traditionally been used to treat itch. The quality of ACTPER as a research agent was controlled in our experiment by cell-based bioassays, as well as by high-performance liquid chromatography (HPLC), using two chemical markers. In the acetone-induced dry skin mice model, the oral administration of ACTPER alleviated dry skin-related skin properties and itching behavior. The RNA and protein expression of the filament aggregating protein (filaggrin) gene, a key factor involved in the regulation of skin barrier function, was significantly increased, as measured by quantitative reverse transcription polymerase chain reaction (RT-PCR) and immunofluorescence assay. To understand the underlying mechanism(s) at the molecular level, HaCaT cells, a human keratinocyte-derived cell line, were treated with various concentrations of ACTPER. We found that the protein expression of filaggrin was indeed upregulated by ACTPER in a dose dependent manner. Data from experiments involving the reporter plasmid containing the xenobiotic response element (XRE), and the chemical antagonist for the aryl hydrocarbon receptor (AhR), indicated that the ACTPER-mediated upregulation of filaggrin was controlled through the activation of the AhR signaling pathway. The molecular docking simulation study predicted that ACTPER might contain chemical compounds that bind directly to AhR. Taken together, our results suggest that ACTPER may provide the platform, based upon which a variety of safe and effective therapeutic agents can be developed to treat itch.

## 1. Introduction

Itching (pruritus) is an uncomfortable sensation of the skin that triggers the urge to scratch [1]. It is one of the primary symptoms of skin diseases, such as atopic dermatitis and psoriasis [2]. 

Although this condition itch is a common symptom experienced by many individuals at some point in their lives, chronic itch caused by various diseases can result in serious insomnia, nervousness, depression, and a dramatically-reduced quality of life [3]. Research into various mechanisms of action has revealed that itching is induced mainly by various pruritogens in the pruriceptors located in the C nerve fibers of peripheral sensory nerves [4]. The transient receptor potential (TRP) channels are a group of ion channels which are mainly expressed in primary afferent sensory neurons [5]. These channels have been shown to play an important role(s) in evoking the itch sensation resulting from the exposure to pruritogens such as histamine, 5-hydroxytryptamine (5-HT) and thymic stromal lymphopoietin (TSLP) [6,7]. Because histamine is a well-known pruritogen, various antihistamines, such as H1 receptor antagonists, are used as primary prescriptions for itch [8]. However, antihistamines are reported to be ineffective for addressing itch caused by dry skin, which is present specifically in patients with atopic dermatitis, psoriasis, and uremic pruritus [9]. As such, the unmet need for antihistamine-resistant itch treatments is very high.

Dry skin is known to be a major cause of symptoms such as skin keratinization and chronic itch [10]. The skin’s moisture content is drastically reduced, due to the diminished water-holding capacity of the epidermis. In a recent series of studies, it is found that dry skin is caused by abnormalities in the “skin barrier” function of the stratum corneum (SC) site [11]. The skin barrier is the outermost layer of the epidermis that protects against harmful external substances, and prevents any evaporation of the skin’s moisture [12]. The skin barrier consists of a combination of fibrous structural proteins like collagen and keratin, and filament-associated proteins including filaggrin, involucrin, and loricrin [13]. Among these, filaggrin is the most important element that maintains the skin barrier function [13]. Recent studies reveal a significant decline in the expression of filaggrin in the skin tissues of both atopic dermatitis and psoriasis mouse models [14,15,16]. Furthermore, in experiments involving filaggrin-knockout mice, transepidermal water loss (TEWL) is severely deteriorated in the skin [17]. Therefore, an effective treatment for dry skin that works by improving the expression of filaggrin could potentially be developed as a therapeutic agent for any antihistamine-resistant itch.

Aryl hydrocarbon receptor (AhR) is a transcription factor that is activated by various xenobiotic ligands, including plant-derived phytochemicals [18]. It has previously been shown that AhR is a major player in the regulation of the adaptive immune system, inhibiting Th17 differentiation and promoting Treg differentiation [19]. Additionally, in a recent series of studies, AhR is found to play an important role in maintaining the skin barrier function [20]. In AhR-knockout mice, the level of TEWL is significantly increased, while the level of skin barrier-related gene expression is reduced in the mice’s back skin [21]. Similarly, it is also reported that AhR improves the function of the skin barrier by activating the expression of filaggrin [22], and that there is a significant increase in dry skin phenotypes in atopic dermatitis patients with AhR polymorphism [23]. Based on these results, studies on AhR-activating agents that may improve the function of the skin barrier through the regulation of filaggrin expression have been actively carried out, specifically using botanical extracts from *Artemisia princeps* and *Rhodiola crenulata* [24,25].

*Actinidia arguta* (Siebold & Zucc.) Planch. ex Miq., an edible fruit belonging to the genus Kiwifruit, is widely prescribed in Korea for a variety of inflammatory and allergic diseases. We previously reported that PG102 could effectively ameliorate pathologic conditions in various allergic disease mouse models, including atopic dermatitis, rhinitis, asthma and food allergies by regulating the expression levels of Th1 and Th2 cytokines [26,27,28,29,30]. Consistent with these data, the treating with PG102 of 90 individuals who had abnormally high levels of IgE (>300 IU/mL), significantly reduced the serum levels of IgE, ECP, TARC, IL-4, and IL-5, compared with the placebo group [31]. Based on these results, PG102 has been approved as a nutraceutical in Korea.

*Perilla frutescens* (L.) Britton, an edible plant belonging to the family Labiatae, has been traditionally used to treat itch and allergic diseases, owing to its sedative and analgesic effects. Furthermore, the active compound of *Perilla frutescens*, luteolin, has been reported to have anti-pruritic effects by inhibiting the histamine secretion in the serotonin-induced itch mice model [32]. Therefore, we hypothesize that the combination of *Actinidia arguta* and *Perilla frutescens* might successfully ameliorate itch.

In this study, we developed a formulation called ACTPER (also called PG102P), a water-soluble extract from a mixture of *Actinidia arguta* and *Perilla frutescens*. This formulation was based on the assumption that the combined use of these two plants might have additive or synergistic effects upon itch. When tested in an acetone-induced dry skin mice model, ACTPER significantly ameliorated dry skin-induced itch via the upregulation of filaggrin expression. Data from experiments involving HaCaT cells suggest that filaggrin gene expression is upregulated by ACTPER through the control of AhR. These results suggest that ACTPER may be a potent and safe agent in the alleviation of dry skin-induced itch.

## 2. Materials and Methods

### 2.1. Preparation of Water-Soluble Extracts from Plants

*Actinidia arguta* was purchased from a company specializing in this fruit (Hurst’s Berry Farm, McMinnville, OR, USA) and *Perilla frutescens* was purchased from Humanherb Co., Ltd. (Gyeongsan, Korea). Both were identified by the Plant DNA Bank in Korea (Seoul, Korea) using their genome sequences. Three different extracts were prepared from *A. arguta*, *P. frutescens*, or the combination of both plants. In the case of *A. arguta*, only fruit parts were used, while for *P. frutescens*, only leaves were taken. These plants parts were extracted, individually or in mixture, by heating in distilled water (DW) for three hours, followed by filtration (No. 2; 110 mm, Whatman International Ltd., Kent, UK). The filtered extract was concentrated by a rotary evaporator and lyophilized. This process generated brown powder. The extraction yield was calculated as the weight ratio of the final lyophilized powder to the dried raw plant material used for the extraction. The yield was about 40%, 10% and 45% for *A. arguta* alone, *P. frutescens* alone, and the combination of both plants, respectively.

### 2.2. High-Performance Liquid Chromatography (HPLC) Analysis

For the qualitative and quantitative analyses of ACTPER, standard solutions of hydroxymethylfurfural, 2-furoic acid, protocatechuic acid, chlorogenic acid, caffeic acid, hyperoside, apigenin 7-O glucuronide and rosmarinic acid were prepared by dissolving the reference compounds in methanol separately. Sample solutions for analysis were prepared by dissolving ACTPER and an extract of each plant (*Actinidia arguta* and *Perilla frutescens*) in water at the concentration of 20 mg/mL. All samples for analysis were filtered through a 0.45 μm membrane filter.

Analytical samples were studied by HPLC-PDA (Waters, Millford, MA, USA) with an Atlantis T3 column (4.6 mm × 250 mm, 5 μm, Waters, Millford, MA, USA). The mobile phase was composed of A (Water containing 0.01% trifluoroacetic acid) and B (Acetonitrile containing 0.01% trifluoroacetic acid) with a gradient elution: 5% B (0–5 min), 5–20% B (5–25 min), 20–30% B (25–40 min), 30–45% B (40–50 min), 45–100% B (50–55 min); then keeping 100% B for 5 min to clean the column, and the re-equilibrating step of the column was 5% B isocratic for 5 min. The flow rate was 1.0 mL/min, and the injection volume was 10 μL. The samples were analyzed at a wavelength of 254 nm and the column temperature was maintained at 25 °C.

### 2.3. Experimental Animals

All experimental procedures were conducted in compliance with the guidelines set by the Institutional Animal Care and Use Committee of Seoul National University. Male ICR mice at five weeks old were purchased from Orientbio Inc. (Seongnam, Korea), and housed in an air-conditioned facility at Seoul National University under a fixed 12 h light/dark cycle. All animal experiments were carried out in accordance with the Guide for Animal Experimentation of Seoul National University. The protocol was approved by the Institutional Animal Care and Use Committee of Seoul National University (Approval Number: SNU-150825-4).

### 2.4. Acetone-Induced Dry Skin Mice Model

Acetone-induced dry skin was generated as described previously [33]. Briefly, 5-wk male ICR mice were topically administered with a 6 × 6 cm acetone (Sigma, St. Louis, MO, USA) soaked cotton pad for 3 consecutive days. From day 0, applicable doses of respective extracts (200 mg/kg for *A. arguta* or *P. frutescens* extract; 100, 200, 400 mg/kg for ACTPER) were orally administered on a daily basis. No behavioral alterations were observed during the treatment with the plant extracts. The vehicle group was orally administered distilled water as a vehicle. Ten minutes after the last exposure to acetone, scratch bouts were measured as the number of times that the animals scratched the treated site with the hind paw over 30 min.

### 2.5. Measurement of Skin Properties

The skin properties of mice back skin were measured as described previously [33]. Transepidermal water loss (TEWL) and stratum corneum (SC) hydration were all measured by non-invasive techniques on anaesthetized animals. Ten minutes after the last exposure to acetone, TEWL was determined using a Tewameter TM300 (Courage + Khazaka GmbH, Cologne, Germany) and expressed as g/m^2^/h, while stratum corneum hydration was measured by a Corneometer CM825 (Courage + Khazaka Electronic GmbH, Cologne, Germany) according to the manufacturer’s protocol. Three measurements were obtained at each time point, and the mean value expressed for each mouse.

### 2.6. Immunofluorescence Assay

Mice skin tissues were formalin fixed and embedded in paraffin. Tissue sections (5 μm) were deparaffinized using Xylene, and rehydrated in a graded ethanol series. Following heat-induced citrate-base antigen unmasking (pH 6.0, Vector Laboratories, Burlingame, CA, USA), blocking was performed with 0.1% BSA in PBS for 2 h at room temperature. Tissue sections were incubated with a polyclonal rabbit anti-filaggrin antibody (Cat. No. 905804, Biolegend, San Diego, CA, USA) overnight at 4 °C, and with the secondary goat anti-rabbit IgG H&L (Alexa Fluor 488, Abcam, Cambridge, UK) for 1 h at room temperature. For a negative control of the nonspecific fluorescent signal, tissue sections were incubated only with the secondary antibody (omitting the primary antibody step). Slides were visualized through an Olympus BX60 fluorescence microscope (Olympus, Tokyo, Japan) and captured with an Olympus DP71 camera (Olympus, Tokyo, Japan) using DP manager image acquisition software (version 03.03, Olympus, Tokyo, Japan). The relative fluorescence intensity between the epidermis and the epidermis-dermis cross section was measured using ImageJ (version 1.50i, National Institutes of Health, Bethesda, MD, USA).

### 2.7. Quantitative Reverse Transcription Polymerase Chain Reaction (qRT-PCR) Analysis

The back skin tissues of mice were cut and kept at −70 °C. HaCaT cells were plated in 6-well cell culture plates. Twenty-four hours later, these cells were treated with 0.25, 0.5, 1, 2 mg/mL of ACTPER for 48 h. Total RNA from mice back skin or HaCaT cells was isolated using TRIzol reagent (Invitrogen, Carlsbad, CA, USA) following the manufacturer’s instructions. The amount and purity of the RNA was measured using the Nanodrop 2000 spectrophotometer (ThermoFisher, Waltham, MA, USA). One microgram of RNA was converted to cDNA using an oligo-dT primer (Qiagen, Valencia, CA) and AMV reverse transcriptase (TaKaRa, Shiga, Japan). One microgram of this cDNA per sample was used for a quantitative polymerase chain reaction using SYBR Premix Ex TaqTM (TaKaRa, Shiga, Japan). Conditions for PCR were denaturation at 95 °C for 5 s, annealing and extension at 60 °C for 20 s. The primer sequences used in this study were [forward, GAATCCATATTTACAGCAAAGCACCTTG; reverse, GGTATGTCCAATGTGATTGCACGATTG] for mouse Filaggrin, [forward, ACTCCTGGTGCTGCTGTTTT; reverse, GATATGGCAGGGGATCAGAA] for mouse Involucrin, [forward, GAGGTCTTTCCACAACCCAC; reverse, TCCCTCACTCATCTTCCCTG] for mouse Loricrin, [forward, CTGTCCCTGTATGCCTCTG; reverse, ATGTCACGCACGATTTCC] for mouse beta-actin, [forward, TCTGAAGAACCCAGATGATCCA; reverse, CATCAAAAGAAACTCAGTAAAGTCCAA] for human Filaggrin, [forward, CAGCAGTCATGTGCTTTTCCT; reverse, TCCTCCAGTCAATACCCATCAG] for human Involucrin, [forward, GAGTTGGAGGTGTTTTCCAGGG; reverse, GCAGAACTAGATGCAGCCGGA] for human Loricrin, and [forward, ACAGCCTGGATAGCAACG; reverse, CACCAACTGGGACGACAT] for human beta-actin. RNA levels were normalized by the level of beta-actin and the relative changes in gene expression were measured using the 2^−∆∆Ct^ method.

### 2.8. Cell Culture and Reagents

The human keratinocyte cell line HaCaT was purchased from CLS Cell Lines Service GmbH (Eppelheim, Germany) and tested for mycoplasma contamination using a PCR Mycoplasma Test Kit I/C (PromoCell, Heidelberg, Germany) according to the manufacturer’s protocol. Cells were cultured in Dulbecco’s modified Eagle’s medium (ThermoFisher, Waltham, MA, USA) containing 10% fetal bovine serum (FBS, Corning, NY, USA) and antibiotics (100 U/mL penicillin and 100 μg/mL streptomycin) at 37 °C under 5% CO_2_. In all experiments, cells at 70% confluence were used to avoid unexpected differentiation. CH233191 was purchased from Sigma (St Louis, MO, USA).

### 2.9. Western Blot Analysis

HaCaT cells were plated in 100mm culture dishes. Twenty-four hours later, the cells were treated with 0.25, 0.5, 1, 2 mg/mL of ACTPER for 72 h. After treatment, these same cells were washed with cold PBS and lysed using a phosphosafe extraction buffer (Novagen, Madison, WI, USA). Total protein contents in the cell lysates were determined by a Bradford assay kit (Abcam, Cambridge, UK) according to the manufacturer’s protocol. After reconstituting in the sample buffer, 10 micrograms of protein samples were subjected to SDS-PAGE on Bolt™ 10% Bis-Tris Plus Gels (ThermoFisher, Waltham, MA, USA), and electrophoretically transferred to PVDF membranes (Millipore, Burlington, MA, USA). The membranes were reacted with primary antibodies against filament aggregating protein (Filaggrin) (sc-80609, 1:500, Santa Cruz Biotechnology, Santa Cruz, CA, USA), and β-actin (A5441, 1:5000, Sigma). Membranes were then incubated with HRP-conjugated anti-mouse or anti-rabbit IgG (1:100,000, Sigma) and visualized in films using ECL solution (Millipore, Billerica, MA, USA).

### 2.10. Luciferase Reporter Plasmid Assay

Luciferase reporter plasmid containing the inducible xenobiotic response element (XRE), which directly interact with the aryl hydrocarbon receptor (AhR), was purchased from QIAGEN (Valencia, CA, USA). A reporter plasmid assay was conducted, as described previously [34,35]. Briefly, HaCaT cells were transiently transfected with an XRE-reporter plasmid and a β-galactosidase plasmid (Invitrogen, Carlsbad, CA, USA), using lipofectamine 2000 (Invitrogen, Carlsbad, CA, USA) following the manufacturer’s protocol. Twenty-four hours later, the cells were treated with various concentrations of ACTPER for 6 h. Cell lysates were prepared, and a luciferase activity was measured using the Luciferase Reporter kit (Promega, Madison, WI, USA) with a microplate luminometer (MicroLumatPlus LB96V, Berthold, Germany). Luciferase activity was normalized to β-gal activity.

### 2.11. Molecular Docking Simulation

The molecular docking simulation was carried out by Glide (Schrödinger, New York, NY, USA). The grid for the human AhR structure was generated using a grid-generation module of Glide. The scaling factor of the van der Waals radii was set as 0.8, with the partial charge cutoff as 0.15 for default settings. The binding site of AhR was included in the grid generation. Selected compounds were drawn and optimized by 2D sketcher and MacroModel (Schrödinger, New York, NY, USA), respectively. All possible ionization states and stereoisomer structures of the ligands were generated using the Ionizer option in LigPrep (Schrödinger, New York, NY, USA). While performing the docking of these compounds to AhR, five poses per ligand, were produced by the SP mode of Glide, respectively. The ligand interaction diagram module of Glide was used to analyze ligand-protein interactions.

### 2.12. Calculation of Binding Energy

The ligand binding free energies were computed using the Prime molecular mechanics-based generalized born/surface area (MM-GBSA) model of the Schrödinger suite. The binding free energy (∆G_bind_) was calculated as:

∆G_bind_ = ∆E_MM_ + ∆G_solv_ + ∆G_SA_ (1)

where ∆E_MM_ is the difference in the minimized energies between the AhR-ligand complex and the sum of the energies of the free AhR and the ligand. ∆G_solv_ is the difference between the GBSA solvation energies of the AhR-ligand complex and the sum of the solvation energies of free AhR and ligand. ∆G_SA_ is the difference between the surface area energies of the complex and the sum of the surface area energies of the free AhR and ligand.

### 2.13. Statistical Analysis

All quantitative data were presented as the mean ± standard error of the mean (S.E.M.) from three independent experiments. Differences between the two groups were statistically analyzed using Student’s t-test, whereas one-way Analysis of Variance (ANOVA) was used for multiple comparisons. Data analysis was performed using the GraphPad Prism 8.0 (GraphPad Software, San Diego, CA, USA). Data was considered statistically significant if the *p*-value was less than 0.05.

## 3. Results

### 3.1. Quality of ACTPER is Measured by HPLC and Cell-Based Bioassay

**Figure 1:**
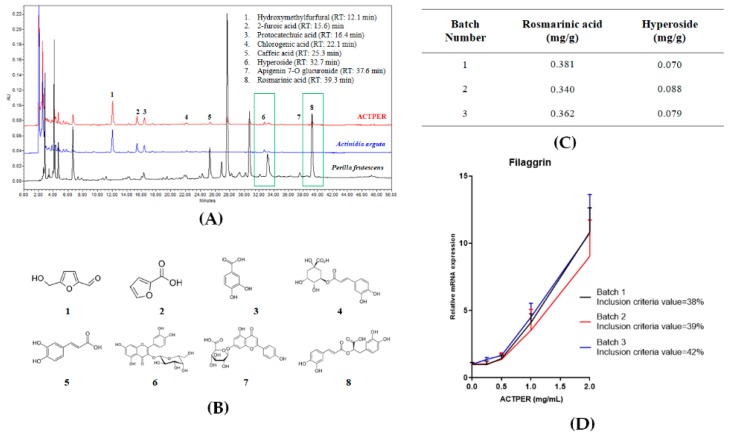
Description of the quality control of ACTPER. (**A**) Representative high-performance liquid chromatography (HPLC) chromatogram of ACTPER; (**B**) Molecular structure of chemical compounds contained in ACTPER; (**C**) Quantification of marker compounds hyperoside and rosmarinic acid; (**D**) Cell-based bioassay using HaCaT cells. Values represent the mean ± S.E.M. of three independent experiments.

To establish the batch-to-batch consistency of ACTPER, the contents of two marker compounds, rosmarinic acid and hyperoside, were quantified using HPLC (Figure 1A,B), and only the extracts containing these compounds within the set range (0.33–0.39 mg/g for rosmarinic acid and 0.06–1.02 mg/g for hyperoside) were used for this study (Figure 1C).

In addition, we conducted a cell-based bioassay using filaggrin, a retrospectively selected protein biomarker that regulates the maintenance of the skin barrier function (see below). HaCaT cells were treated with ACTPER at different concentrations for 48 h, and the RNA level of filaggrin was measured by quantitative RT-PCR. When the cells were treated with ACTPER, filaggrin expression was increased in a dose-dependent manner (Figure 1D). When batches were prepared at different times, those chosen for research were only the ones whose 1 mg/mL ACTPER showed bioactivity in the range of 35–45% of the bioactivity of the 2 mg/mL ACTPER from the same batch, assuming the effect of 2mg/mL on the RNA level of filaggrin to be 100% activity. All batches of ACTPER satisfied the inclusion criteria.

### 3.2. The Combined Use of Actinidia arguta and Perilla frutescens Synergistically Improves Acetone-Induced Itch in Mice

**Figure 2:**
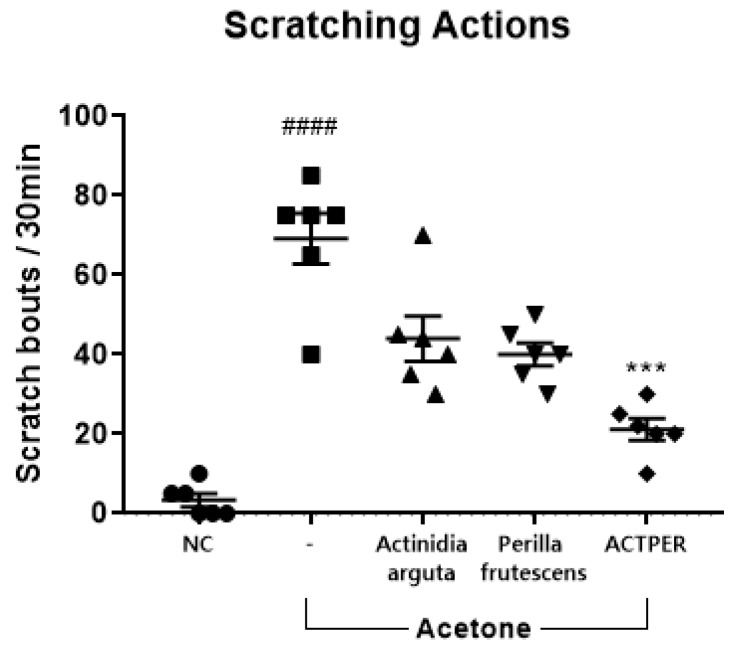
Anti-pruritic effects of *Actinidia arguta*, *Perilla frutescens* and ACTPER in acetone-treated mice. Effects of water-soluble extracts from *Actinidia arguta*, *Perilla frutescens*, and ACTPER (200 mg/kg) on acetone-induced itch were determined as described in the Materials and Methods section. *n* = 6 per group. All data are shown as mean ± S.E.M. ^####^ *p* < 0.0001 compared with control group; *** *p* < 0.001 compared with vehicle group.

Since acetone-induced dry skin is known to cause itching behavior in mice [33,36], this model was used to test the effect of three water-soluble extracts (from *Actinidia arguta*, *Perilla frutescens* or the combination of these two plants, respectively) on dry skin-induced itch. As shown in Figure 2, the number of scratching actions increased drastically in the acetone-treated groups, from virtually none to 69 ± 6. When treated with an extract from an individual plant, there appeared to be a positive trend: The scratching frequency was lowered to 44 ± 7 for *A. arguta*, and to 40 ± 4 for *P. frutescens*. However, there was no statistical significance. When administered with the extract of both plants, however, the number of scratching incidences dropped sharply to 21 ± 3, by almost 70%, in a statistically significant manner, indicating that the simultaneous use of two plants had a synergistic effect on dry skin-induced itch.

### 3.3. ACTPER Ameliorates Dry Skin-Induced Itch in Acetone-Treated Mice

**Figure 3:**
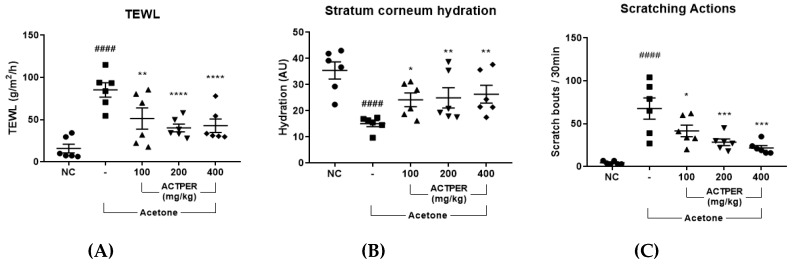
ACTPER alleviates dry-skin induced itch in acetone-treated mice. Effect of ACTPER on (**A**) Transepidermal water loss (TEWL), (**B**) Stratum corneum (SC) hydration and (**C**) scratching behaviors. *n* = 6 per group. All data are shown as mean ± S.E.M. ^####^ *p* < 0.0001 compared with control group; * *p* < 0.05, ** *p* < 0.01, *** *p* < 0.001, **** *p* < 0.0001 compared with vehicle group.

We investigated the effects of ACTPER on dry skin in acetone-treated mice. As shown in Figure 3A, the application of acetone caused a significant increase in TEWL, and this effect was reduced upon ACTPER treatment in a dose-dependent manner. Similarly, ACTPER treatment improved the hydration level of the stratum corneum, which had been reduced by acetone (Figure 3B). These data indicate that ACTPER might alleviate dry skin in acetone-treated mice.

Next, we tested whether ACTPER administration affected the number of scratching actions in acetone-treated mice. As shown in Figure 3C, the number of measured actions increased sharply in the acetone-treated group. When treated with ACTPER, however, this itching behavior was markedly reduced in a dose-dependent manner (Figure 3C, the representative videos of the scratching actions are shown in Supplementary Materials), indicating that ACTPER may ameliorate dry-skin induced itch.

### 3.4. ACTPER Promotes the Expression Level of Filaggrin in Acetone-Treated Mice Back Skin

**Figure 4:**
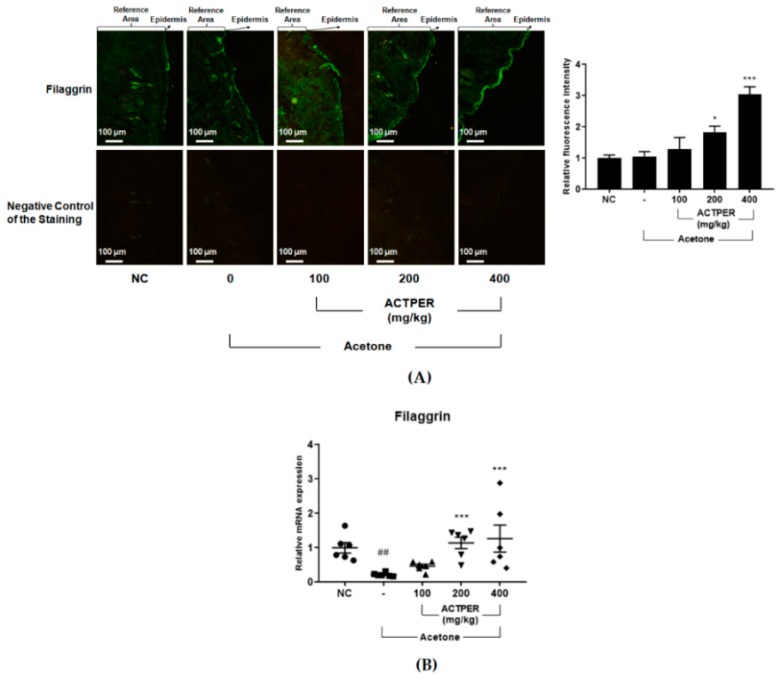
ACTPER promotes the expression of filament aggregating protein (filaggrin) in the acetone-treated skin of the backs of mice. (**A**) IFA staining for filaggrin in the skin of the mice’s backs; (**B**) RNA level of filaggrin in mice back skin. *n* = 6 per group. All data are shown as mean ± S.E.M. ^##^ *p* < 0.01 compared with control group; * *p* < 0.05, *** *p* < 0.001 compared with vehicle group.

Since filaggrin is one of the key players needed for the maintenance of skin barrier function [13], we investigated ACTPER’s effect on this protein. It was clear that the ACTPER treatment promoted the expression level of filaggrin, as compared to acetone-treated mice in a dose-dependent manner (Figure 4A).

Next, we assessed whether ACTPER could regulate the expression of other skin barrier-related genes such as involucrin and loricrin. As shown in Figure 4B, the RNA level of filaggrin in the back skin of mice treated with acetone was significantly reduced, and this effect was diminished upon treatment with ACTPER in a dose-dependent manner. Interestingly, no change in the RNA level of involucrin and loricrin was observed (Figure S1A,B). Taken together, these data suggest that ACTPER might improve skin barrier function, specifically by upregulating filaggrin gene expression.

### 3.5. ACTPER Promotes the Expression of Filaggrin in HaCaT cells

**Figure 5:**
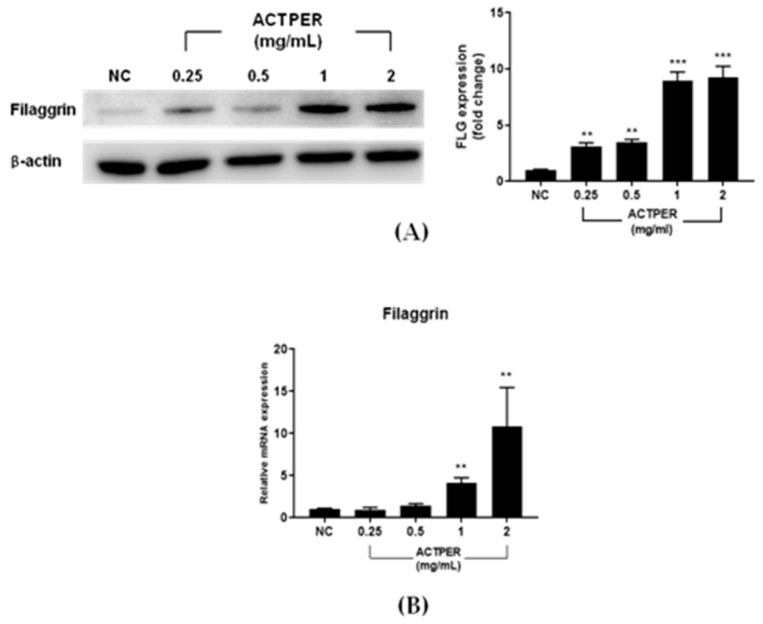
ACTPER promotes the expression of filaggrin in HaCaT cells. (**A**) Changes in the level of filaggrin protein; (**B**) Changes in the RNA level of filaggrin. Values represent the mean ± S.E.M. of three independent experiments. ** *p* < 0.01, *** *p* < 0.001 compared with control group.

To understand the mechanism underlying the ACTPER-mediated upregulation of filaggrin, we used HaCaT cells, a human keratinocyte-derived cell line. As clearly shown in Figure 5A, ACTPER treatment increases the protein level of filaggrin in a dose-dependent manner.

Next, we tested whether ACTPER controls the expression of skin barrier-related genes at the transcriptional level. Consistent with protein results, ACTPER effectively increases the RNA level of filaggrin (Figure 5B). However, the expression of involucrin and loricrin was not changed upon ACTPER treatment (Figure S2A,B). Taken together, these data indicate that ACTPER might be a potent positive regulator of filaggrin expression at the RNA level in HaCaT cells.

### 3.6. Effect of ACTPER on Filaggrin Expression was Mediated by the AhR Signaling Pathway

**Figure 6:**
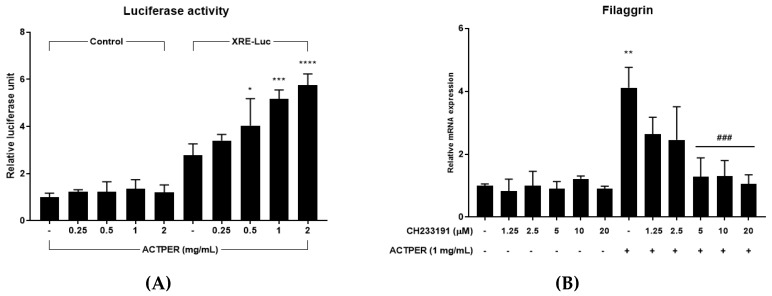
Effects of ACTPER on filaggrin expression were mediated by the aryl hydrocarbon receptor (AhR) signaling pathway. (**A**) Luciferase activity was measured; (**B**) Changes in the RNA level of filaggrin. Values represent the mean ± S.E.M. of three independent experiments. * *p* < 0.05, ** *p* < 0.01, *** *p* < 0.001, **** *p* < 0.0001 compared with control group; ^###^ *p* < 0.001 compared with ACTPER treated group.

Since AhR is well-known to regulate the expression of filaggrin, we tested whether ACTPER could control the AhR signaling pathway using a luciferase reporter plasmid. When cells were treated with ACTPER, the level of luciferase activity gradually increased as the concentration of ACTPER increased (Figure 6A). This result indicates that ACTPER might somehow activate AhR, which subsequently binds to XRE to drive the expression of various genes.

To confirm the relationship between AhR and ACTPER, AhR specific antagonist CH233191 was used. Consistent with the above results, ACTPER increased the RNA level of filaggrin by approximately 4-fold, but treatment with the AhR antagonist reduced the effects of ACTPER in a dose-dependent manner to a virtually basal level at 20 μM CH233191 (Figure 6B). Taken together, these data suggest that filaggrin gene expression is upregulated by ACTPER through the control of AhR.

### 3.7. ACTPER was Predicted to Contain Chemical Compounds Expected to Bind to AhR by Molecular Docking Simulation

**Figure 7:**
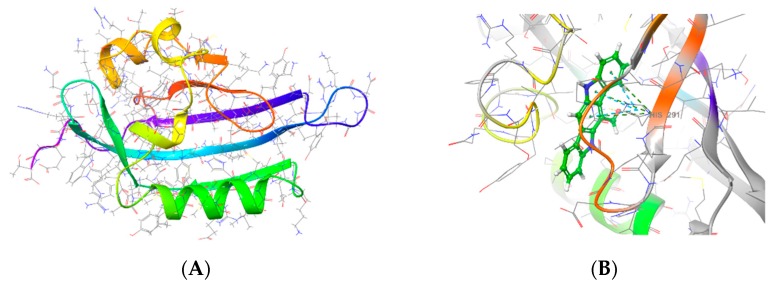

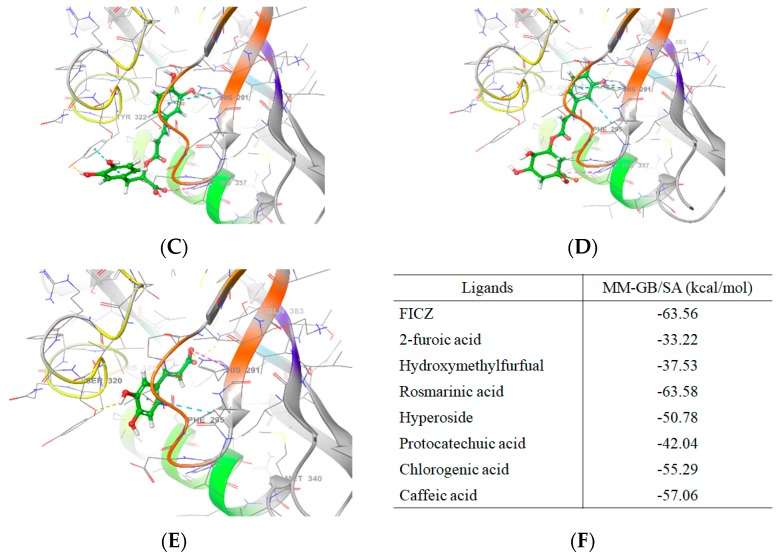
Predicted binding poses and MM-GB/SA values by the molecular docking simulation. ACTPER contains chemical compounds predicted to bind to AhR when analyzed by a molecular docking simulation. (**A**) Crystal structure of AhR-LBD. 2D AhR-LBD interaction diagram in complex with (**B**) FICZ, (**C**) rosmarinic acid, (**D**) chlorogenic acid and (**E**) caffeic acid; (**F**) MM-GBSA binding free energy values. The more negative MM-GBSA value indicates stronger binding.

The above data suggest that some component(s) present in ACTPER might directly interact with AhR as in the case of FICZ, a well-known AhR agonist. To study this possibility, a molecular docking simulation was performed. The structures of the AhR ligand binding domain from *Homo sapiens* was built by homology modeling (Figure 7A), and potential binding poses with FICZ, an AhR agonist, and chemical compounds identified during HPLC analysis as summarized in Figure 1, were generated using Glide software (Figure 7B–E). Next, the MM-GBSA binding free energy values in the simulated binding pose were calculated to forecast the binding affinity between each chemical compound and AhR. As summarized in Figure 7F, the binding affinity of rosmarinic acid to AhR is expected to be very high, as its binding free energy value was very similar to that of FICZ. In addition, caffeic acid, chlorogenic acid and hyperoside were also predicted to have sufficiently high levels of MM-GBSA binding free energy to bind to AhR (Figure 7F). These data indicated that ACTPER might indeed contain chemical compounds that directly bind to AhR.

## 4. Discussion

In this study we showed that an oral administration of the botanical formulation ACTPER could efficiently ameliorate dry skin-induced itch by regulating the expression of filaggrin via the AhR signaling pathway. We also showed that, based on the molecular docking simulation technique, four compounds present in ACTPER (rosmarinic acid, caffeic acid, chlorogenic acid and hyperoside) might be the key molecules involved in this process.

The skin barrier is the outermost epidermal layer that prevents the evaporation of moisture [11]. Skin barrier damage has been reported to cause dry skin, which may result in chronic itch [10]. It is recently reported that the skin barrier function could be seriously compromised in patients with atopic dermatitis, psoriasis and uremic pruritus, which all have dry skin as a characteristic symptom. Dysregulation of the skin barrier function in these diseases was manifested by filaggrin defection [13,37,38,39,40,41,42]. Therefore, ACTPER, which may fundamentally improve the skin barrier function by regulating filaggrin expression, could be developed as a therapeutic for chronic itch.

There are three major structural proteins consisting of skin barriers: Filaggrin, involucrin and loricrin. One of the most interesting observations made in this study is that ACTPER acts specifically on filaggrin. Data from experiments involving the XRE containing reporter plasmids and a specific chemical antagonist of AhR demonstrated that ACTPER regulated the expression of filaggrin through AhR. This is consistent with a previous report showing that solely filaggrin expression is affected by this receptor, while the expression of the two other genes remains unchanged [43]. It is recently shown that the RNA level of filaggrin—but not of involucrin or loricrin—is increased when mice are treated with chemical DHA—a dual antagonist for PPARα and PPARγ, both well-known transcription factors involved in adipocyte differentiation [44]. It may be fruitful to test whether the expression of filaggrin is affected by PPAR signaling pathways independent of or through cross-talking with AhR signaling.

ACTPER may work, on not only dry skin, but also against pruritogen-induced itch. We have previously shown that an extract from *A. arguta* called PG102, one of two plants used for making ACTPER, could effectively down-regulate the expression of IL-4 [29,30]. IL-31 is a well-known pruritogen produced from Th2 cells [45,46]; for example, data from phase II trials involving an anti-IL-31 receptor humanized antibody showed that inhibiting the IL-31 activities could indeed improve pruritic VAS scores [47]. Since the expression of Th2 cytokines is generally controlled in a set, it may be possible that ACTPER reduces IL-31 levels [48]. Taken together, ACTPER may be developed as a therapeutic agent also for pruritogen-induced itch. Experiments are underway to verify this possibility.

Data from molecular docking simulation studies suggests the possibility of developing a mixture of small molecules that can control the activity of AhR. Rosmarinic acid is predicted to have a binding free energy value comparable to that of FICZ, a well-known AhR agonist, but it has never been studied in the context of AhR. There are also three other molecules (caffeic acid, chlorogenic acid and hyperoside) with binding free energies similar to that of FICZ. Therefore, it may be possible to reconstitute the effects of ACTPER by combining these molecules. Further studies are warranted to unravel the contribution levels of the respective compounds to the bioactivity shown by ACTPER.

Together with data from a bulk of literatures, the findings of this report strongly suggest that ACTPER could provide a fundamental treatment for skin diseases involving dry skin and itch. The safety levels of *Actinidia arguta* and *Perilla frutescens* are well established, not only by their long historical use, but also by a variety of human and animal studies [26,27,28,31,32,49,50]. Taken together, ACTPER appears to have the potential to be developed as a safe and effective therapeutic agent for different types of skin diseases.

## Supplementary Materials

The following are available online at https://www.mdpi.com/2072-6643/11/6/1366/s1, Figure S1: ACTPER does not regulate the expression of involucrin and loricrin in acetone-treated mice back skin; Figure S2: ACTPER does not promote the expression of involucrin and loricrin in HaCaT cells.
