# Supplementary material for: Water-Soluble Extract from Actinidia arguta (Siebold & Zucc.) Planch. ex Miq. and Perilla frutescens (L.) Britton, ACTPER, Ameliorates a Dry Skin-Induced Itch in a Mice Model and Promotes Filaggrin Expression by Activating the AhR Signaling in HaCaT Cells
Source: Nutrients. 2019 Jun 18;11(6):1366. doi: 10.3390/nu11061366 (PMC6627490; doi:10.3390/nu11061366)
Supplement: Supplementary file 1 [file nutrients-11-01366-s001.pdf]

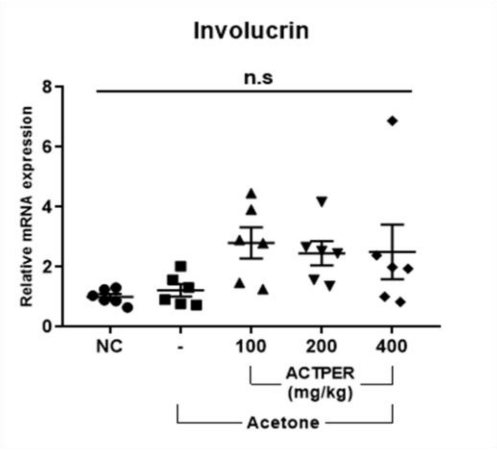

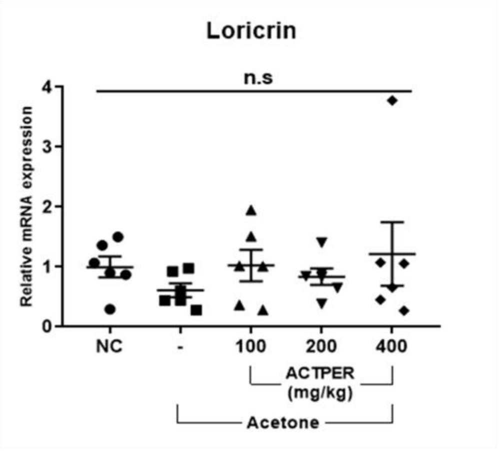

(A) **(B)**

Figure S1. ACTPER does not regulate the expression of involucrin and loricrin in acetone-treated mice back skin. RNA levels of (**A**) involucrin and (**B**) loricrin in mice back skin. n = 6 per group. All data are shown as mean ± S.E.M.

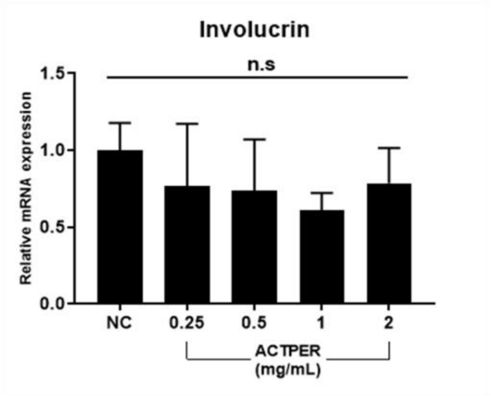

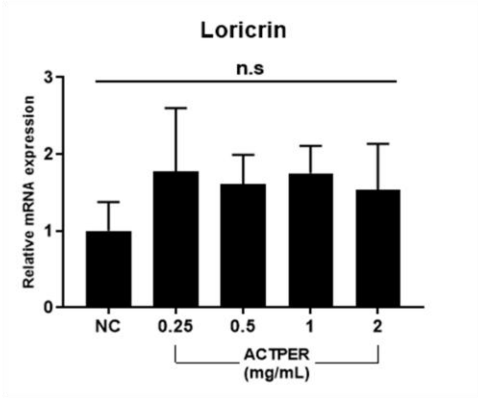

(A) **(B)**

Figure S2. ACTPER does not promote the expression of involucrin and loricrin in HaCaT cells. Changes in the RNA level of (**A**) involucrin and (**B**) loricrin. Values represent the mean ± S.E.M. of three independent experiments.
